# Infection of anopheline mosquitoes with *Wolbachia*: Implications for malaria control

**DOI:** 10.1371/journal.ppat.1007333

**Published:** 2018-11-15

**Authors:** Fabio M. Gomes, Carolina Barillas-Mury

**Affiliations:** Mosquito Immunity and Vector Competence Section, Laboratory of Malaria and Vector Research, National Institute of Allergy and Infectious Diseases, National Institutes of Health, Rockville, Maryland, United States of America; Washington University School of Medicine, UNITED STATES

## Malaria eradication and vector control in Africa

Africa carries a disproportionate share of the malaria burden. For example, more than 90% of the 446,000 malaria-related deaths reported in 2015 occurred in sub-Saharan Africa [1]. *Anopheles gambiae*, *A*. *coluzzii*, *A*. *arabiensis*, and *A*. *funestus* are some of the most important African vectors of malaria [2,3]. Other species, such as *A*. *melas* and *A*. *merus* are also efficient vectors but have a limited geographical distribution [3]. Insecticide-based strategies, mainly the distribution of insecticide-treated nets and indoor residual spraying, are efficient against a wide range of mosquitoes and are the current cornerstones of malaria control programs. However, the growing number of reports of insecticide resistance is driving the development of novel vector control strategies [4].

## *Wolbachia* infection in *Drosophila* and protection against viruses

*Wolbachia* is a genus of vertically transmitted endosymbiotic alphaproteobacteria that infect about 40% of arthropod species [5]. *Wolbachia* infection often reaches a high prevalence in natural insect populations, often through the induction of cytoplasmic incompatibility (CI) [6]. Briefly, CI is a mechanism by which the sperm of *Wolbachia*-infected males is unable to form viable offspring when eggs of uninfected females are fertilized, whereas eggs of infected females are viable. This mechanism allows the spread of *Wolbachia* through the population by giving infected females a reproductive advantage.

Many *Wolbachia* strains are known to confer resistance against viral infections. For example, the *Wolbachia w*Mel strain protects *Drosophila melanogaster* against *Drosophila* C virus (DCV) [7] and Flock House virus [8], suggesting that protection is efficient against a variety of RNA viruses. Although the molecular basis of protection remains under debate, the *Wolbachia* genome has been shown to be a key player. Protection by genetic variants of *w*Mel *D*. *melanogaster* correlates with the phylogeny of the *Wolbachia* strains analyzed [9]. Furthermore, viral protection was observed in *D*. *simulans* lines infected with *Wolbachia* strains phylogenetically related to *w*Mel but not with more divergent strains [10].

## *Wolbachia* in mosquito vectors and its effects on disease transmission

*Wolbachia* also protects mosquitoes from viral infections. Native *w*Pip infections in *Culex quinquefasciastus* increases host resistance to West Nile virus [11]. Similarly, native *Wolbachia* limits dengue virus (DENV) infection in *Aedes albopictus* [12]. However, the effect of *Wolbachia* on viruses is strain and host specific. For example, native *Wolbachia* had no effect on DENV infection in *Aedes notoscriptus* [13].

Transinfection of host with *Wolbachia* triggers stronger antiviral protection than native *Wolbachia* strains [14]. The *Drosophila w*Mel-Pop [15] and *w*Mel [16] *Wolbachia* strains were adapted to infect *Aedes aegypti* cell lines and were used to transinfect *A*. *aegypti* mosquitoes by embryonic microinjection. For both strains, transinfected mosquitoes displayed strong vertical transmission with CI and greatly reduced DENV transmission [16]. *A*. *aegypti* transinfected with *w*Mel were released in test sites in Australia, where they replaced natural mosquito populations and reached near fixation levels within a few months after their initial release [17]. *Wolbachia* prevalence has been stable for several years in these locations [17,18] and has slowly spread throughout the area [19].

A systematic survey in Thailand detected native *Wolbachia* infections in 23 species of mosquitoes, including species from the genera *Aedes*, *Culex*, and *Mansonia* [20]. None of the 19 species of *Anopheles* screened were found to harbor *Wolbachia*. The lack of *Wolbachia* infections in anophelines was later confirmed in screenings using European, African, and American specimens [21,22].

Thoracic microinjections of *Wolbachia* in *A*. *gambiae* resulted in ubiquitous infections in somatic tissues. However, germline cells were not infected and this precluded vertical transmission [23]. A stable *Anopheles stephensi* line infected with *A*. *albopictus w*AlbB strain was established by embryonic microinjection of *A*. *albopictus* ooplasm [24]. *Wolbachia*-infected *A*. *stephensi* were partially protected against *Plasmodium falciparum* infections, resulting in a modest decrease in oocyst numbers and a strong reduction in salivary gland sporozoites. Similar protection against *Plasmodium* was also observed when *w*Mel-infected *A*. *aegypti* were challenged with *P*. *gallinaceum* [15] or when *A*. *gambiae* carrying somatic *Wolbachia* infections were infected with *P*. *falciparum* [23]. Other reports have suggested that some combinations of *Wolbachia*, host, and environmental factors could actually enhance *Plasmodium* infection in mosquitoes [25,26].

## Identification of *Wolbachia* in natural African anopheline mosquito populations

**Fig 1:**
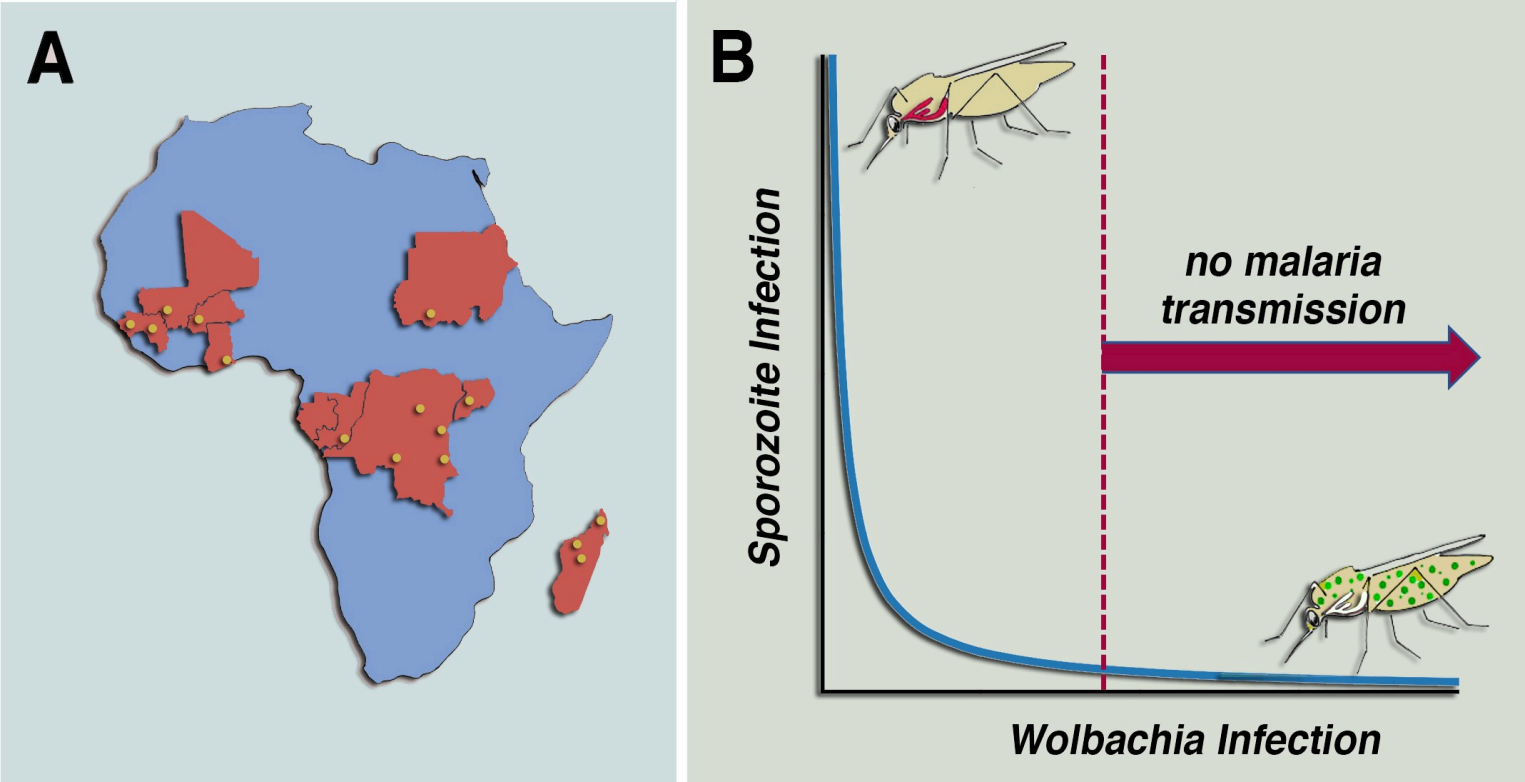
Effect of mosquito infection with native African *Wolbachia* strains on *P*. *falciparum* transmission. (A) Native *Wolbachia* infections were identified in independent field collections of *A*. *gambiae sensu lato* in several countries in sub-Saharan Africa. Approximate locations of field collections, when made available, are indicated as yellow circles. (B) The presence of systemic *Wolbachia* infection reduces *P*. *falciparum* sporozoite infection of mosquito salivary glands. The effect of native *Wolbachia* in *Anopheles* mosquitoes collected in Mali is dose dependent and follows a nonlinear negative correlation. These observations suggest that there is a threshold level of *Wolbachia* infection in mosquitoes above which malaria transmission would no longer be effective.

Recently, traces of *Wolbachia* genomic DNA were identified in a microbiome survey of the reproductive organs of *A*. *gambiae* and *A*. *coluzzii* in malaria-endemic areas of Burkina Faso in West Africa (Fig 1A) [27,28]. *Wolbachia*-specific PCR amplification and sequencing was used to confirm the presence of *Wolbachia* in these mosquito populations [27,29]. In an independent study, native *Wolbachia* infections were identified in the same mosquito species collected in two villages from Mali (Fig 1A) [30]. *Wolbachia* infection was observed in two collections made five years apart, indicating that the symbiosis has remained stable in the population. More recently, native *Wolbachia* infections were identified in a broad range of African anophelines from the Democratic Republic of Congo, Guinea, Uganda, and Madagascar [31], as well as Gabon [32]. Phylogenetic analysis suggested that several independent horizontal transfers of *Wolbachia* infection have occurred, but whole-genome sequencing will be necessary for an in-depth analysis of the evolutionary relationship between these strains.

A significant reduction in *Plasmodium* prevalence was observed in mosquitoes carrying native *Wolbachia* infection in Mali. This effect was dose dependent, with a nonlinear negative correlation (Fig 1B) [29,30]. Because confounding environmental or ecological variables could influence the analysis of field-collected mosquitoes, a colony of *w*Anga-Mali-infected *A*. *coluzzii* was established. Infection of colony-adapted mosquitoes with *P*. *falciparum* NF54 confirmed that *Wolbachia* negatively affects sporozoite infection with a similar negative correlation (Fig 1B) [30].

## Challenges for the development of *Wolbachia* as a tool for malaria control

The identification of native *Wolbachia* infections in *A*. *gambiae* that reduce malaria transmission is a remarkable finding. The fact that several species of *Anopheles*, including all the major malaria vectors in Africa, have been shown to harbor a variety of *Wolbachia* strains opens the possibility that one of these strains may confer CI and disrupt disease transmission. However, several challenges remain before *Wolbachia* can be proposed as a tool for malaria control. Implementation of *Wolbachia-*based strategies would rely on CI for *Wolbachia* to spread rapidly in natural populations following mosquito releases. At present, it is not clear whether native *Wolbachia* can induce CI in *Anopheles*. Induction of CI was not observed in caged experiments using *w*Anga-BF-infected *Anopheles*. However, CI has been shown to be influenced by environmental factors [33], and optimal conditions might be different from the ones typically used for rearing laboratory mosquitoes.

*Wolbachia* levels have also been shown to influence CI [34,35], and the lack of CI might be due to the low levels of native *Wolbachia* infection in *Anopheles* (*w*Anga-Mali genome copies are usually less than 0.1% of the mosquito genome). *Wolbachia* levels are also positively correlated with the intensity of protection against viruses. Similarly, higher *Wolbachia* levels may induce CI in anophelines and confer stronger protection against *Plasmodium*, which will be essential for *Wolbachia* to be developed as a tool against malaria.

The mechanisms limiting *Wolbachia* levels are not well understood. One hypothesis is that *Wolbachia* might have adapted to control its replication as a strategy to hide from host immunity. Alternatively, high levels of *Wolbachia* could reduce mosquito fitness and be negatively selected. Another possibility is that *Anopheles* might not be a good host for *Wolbachia*. For example, the mosquito microbiota limits *Wolbachia* infections in *A*. *gambiae*. More specifically, the presence of bacteria from the genus *Asaia* in germline cells prevents *Wolbachia* invasion of *A*. *gambiae* ovaries [36]. Microbiome analyses of mosquitoes collected from *Wolbachia-*endemic areas found that the prevalence of *Asaia* was lower than in reports from other locations in Africa [28] or failed to find evidence of co-infections between *Wolbachia* and *Asaia* [31]. However, the level of *P*. *falciparum* infection was not significantly different between females with or without *Asaia* in *A*. *gambiae s*.*l*. females collected in Guinea [31]. It is also possible that *Anopheles* metabolism or immunity might limit *Wolbachia* levels. In *Aedes*, *Wolbachia* manipulates host lipid metabolism, and cholesterol sequestration seems to play a role in the protection against viruses [37,38]. The current understanding of *Anopheles*’ lipid metabolism is incipient, but poor nutritional stores could explain the inability of anophelines to sustain high *Wolbachia* densities.

Infection of embryo-derived somatic cell lines would be an invaluable in vitro model to identify the components limiting *w*Anga levels. Transcriptomics and metabolomics of infected cells would also allow for a detailed analysis of the effect of *Wolbachia* on host homeostasis. Once potential target genes are identified, it would be possible to carry out functional screens to identify genes or nutrients limiting *Wolbachia* infection. However, it will still be necessary to evaluate how this information can be translated to a whole mosquito and eventually to the field. An alternative approach would be the development of directed evolution protocols that would allow progressive adaptation of *Wolbachia* or hosts to sustain higher bacteria levels.

## Future perspective

Laboratory transinfection of *Wolbachia* to African vectors of malaria has been limited to somatic tissues, and *Wolbachia* failed to be vertically transmitted. We now know that *Wolbachia* strains in Africa have been able to overcome this barrier and establish infections in anophelines. The presence of this bacteria negatively correlates with *Plasmodium* sporozoite prevalence. This is an exciting new development in the field, but several challenges remain. It is not clear whether those strains can induce CI, which is needed for implementation of *Wolbachia* as a control strategy. Further studies should address this question and identify the factors limiting *Wolbachia* replication in anophelines. A more detailed understanding of the molecular components mediating *Wolbachia* establishment and host adaptation might make it possible to adapt CI-carrying *Wolbachia* strains to infect anophelines or to increase the levels of native *Wolbachia* infections and disrupt malaria transmission.
